# Tissue Is the Issue: The Diagnosis of Butterfly Brain Lesions

**DOI:** 10.7759/cureus.75538

**Published:** 2024-12-11

**Authors:** Jose Valerio, Noe Santiago Rea, Jorge Zumaeta, Benjamin Graham, Penelope Mantilla-Farfan, Roberto Sanchez

**Affiliations:** 1 Neurological Surgery, Palmetto General Hospital, Hialeah, USA; 2 Neurosurgery, Miami Neuroscience Center at Larkin, South Miami, USA; 3 Neurosurgery, Latinoamerica Valerio Foundation, Weston, USA; 4 Neurological Surgery, Latinoamerica Valerio Foundation, Weston, USA; 5 Neurosurgery, Hospital Nacional Arzobispo Loayza, Lima, PER; 6 Vascular, Tumor and Functional Neurosurgery, Hospital Nacional Guillermo Almenara Irigoyen, Lima, PER; 7 Neurosurgical Oncology, Latinoamerica Valerio Foundation, Weston, USA; 8 Pathology, Palmetto General Hospital, Hialeah, USA; 9 Neurology, Palmetto General Hospital, Hialeah, USA

**Keywords:** biopsy, butterfly lesion, demyelinating neurological disorder, multiple sclerosis, neurosurgery

## Abstract

The corpus callosum can reveal a “butterfly” pattern on imaging in various conditions, including glioblastoma, primary central nervous system lymphoma, tumefactive multiple sclerosis, and toxoplasmosis. Early differentiation among these conditions is crucial to avoid aggressive treatments. In one case, a 70-year-old woman with a history of multiple sclerosis experienced a neurological decline. While imaging suggested a high-grade glioma, a biopsy ultimately confirmed the diagnosis of tumefactive multiple sclerosis. The patient showed improvement with steroid therapy. It is essential to distinguish between high-grade gliomas and tumefactive multiple sclerosis, as gliomas typically present acutely while tumefactive multiple sclerosis progresses more slowly. Utilizing advanced imaging techniques and biopsy aids in achieving an accurate diagnosis, thus preventing unnecessary interventions. Additionally, a multidisciplinary approach is vital for optimal management of these conditions.

## Introduction

The anterior interhemispheric commissure consists of white matter fibers, including part of the corpus callosum [1]. The corpus callosum is divided into the following five segments: rostrum, genu, body, isthmus, and splenium [2]. Fibers from the inferior frontal and inferior parietal lobes project and cross through the genu, while those from the parietal lobe cross at the splenium. The remaining fibers cross through the body of the corpus callosum [1]. When the corpus callosum is involved in the pathological lesions, a butterfly pattern is created due to the bilateral hemispheric pattern [1,3].

Even though glioblastoma and primary central nervous system lymphoma (PCNSL) are the most common lesions with a butterfly pattern on axial imaging involving the corpus callosum with extensions into the bilateral cerebral hemispheres, a wide range of pathologies can affect the corpus callosum causing butterfly pattern [4], including tumefactive multiple sclerosis [5-7] and toxoplasmosis [6].

It is important to keep in mind the previously mentioned differential diagnosis so that performing a stereotactic biopsy can define the treatment for these patients without exposing them to management that carries higher morbidity and mortality [8]. For this reason, we present our case, which initially presented as a malignant tumor pathology but was, in fact, a disease for which only medical management was indicated.

## Case presentation

A 70-year-old female with a self-reported history of multiple sclerosis presented to the emergency department two days after a mechanical fall. The patient’s son reported behavioral changes the day after the fall, and by the second day, she was unable to get out of bed. Otherwise, the patient had been healthy. On physical examination, she was somnolent with expressive aphasia, bradypsychia, right-sided hemiparesis, no facial droop, and deep tendon reflexes +2/4 throughout. The Glasgow Coma Scale score was 10/15.

**Figure 1:**
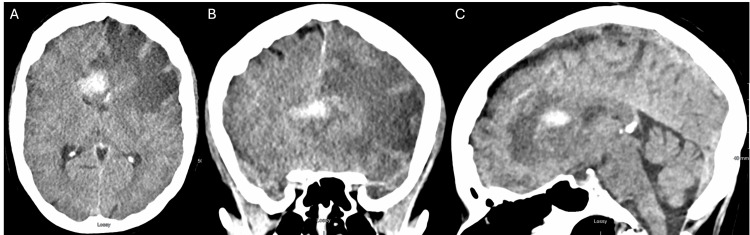
A non-contrast brain CT scan. Axial (A) and coronal (B) views show marked perilesional edema causing a left-ward midline shift. The hemorrhage is located between the body and the genu of the corpus callosum (C).

**Figure 2:**
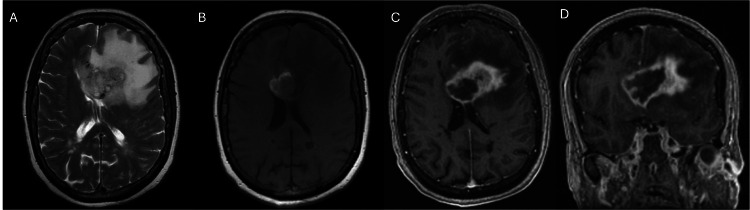
Butterfly-wing lesion on MRI. Axial T2-weighted MRI shows marked perilesional edema collapsing the frontal horns of the lateral ventricles (A). Axial T1-weighted MRI (B) reveals a small hyperintensity around the lesion, which, after contrast administration (C), avidly enhances circumferentially with a non-enhancing center. The coronal gadolinium-enhanced T1-weighted MRI (D) demonstrates the lesion with a necrotic center and peripheral contrast enhancement, crossing both sides of the midline.

Initial imaging included a CT scan of the brain (Figure 1), which revealed a left frontal intra-axial lesion, suggestive of high-grade glioma with surrounding vasogenic edema and central tumoral hemorrhage, causing effacement of the frontal ventricular system horns. A brain MRI with and without contrast (Figure 2) was ordered, and a navigated guided biopsy was performed.

**Figure 3:**
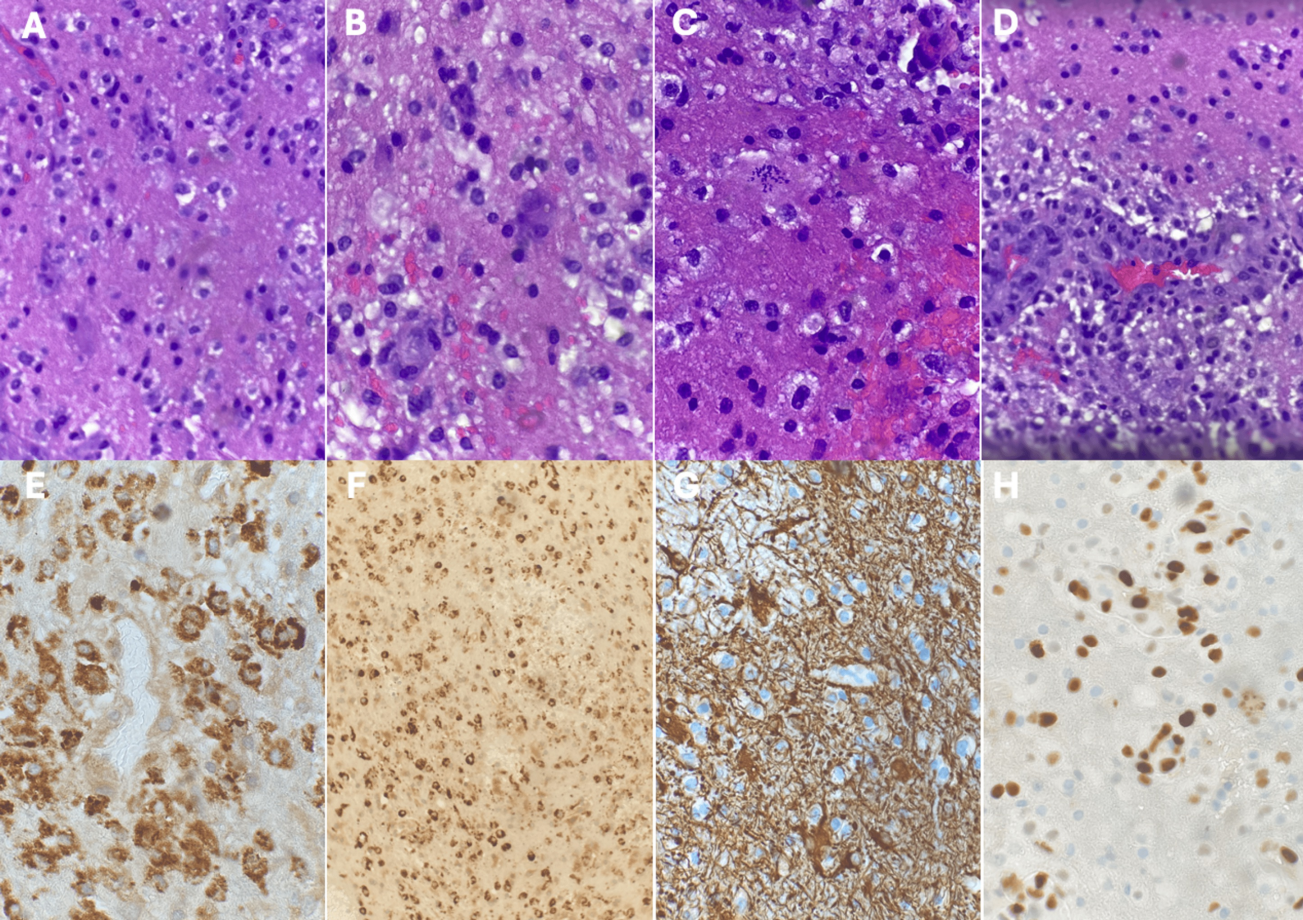
Pathology description. Macrophage-rich lesion demonstrating retained neurofilament with increased foamy macrophages (A). Macrophage-rich lesion with Creutzfeldt cells (B). Macrophage-rich lesion with large “granular mitosis” (C). Vascular proliferation with increased perivascular macrophages (D). CD68 immunohistochemical stain highlighting increased perivascular macrophages (E). CD68 highlights markedly increased macrophages (F). Glial fibrillary acid protein immunohistochemical stain highlighting intact neurofilament and reactive astrocytes without staining of macrophages (blue color) (G). Ki-67 immunohistochemical stain, highlighting increased proliferation index predominantly within macrophages (H).

**Figure 4:**
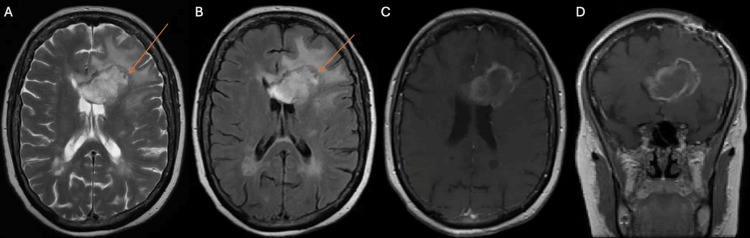
Post-biopsy MRI. Axial T2-weighted MRI shows a hyperintense signal surrounding the bilateral frontal horns, accompanied by evidence of partial subfalcine herniation (A). Axial fluid-attenuated inversion recovery imaging reveals subependymal edema in the ventricular system, with hyperintensity localized to the left centrum semiovale (B). Axial T1-weighted MRI with gadolinium enhancement reveals an irregular lesion in the left frontal lobe (C), resulting in the collapse of the frontal horns on the coronal view (D). In images A and B, a notch from the biopsy can be observed at the periphery of the lesion (orange arrow).

The biopsy results (Figure 3), as confirmed by multiple pathologists from different institutions, were consistent with tumefactive multiple sclerosis, wherein malignant mimickers such as histiocytic sarcoma were ruled out. Although the possibility of lymphoma, such as lymphomatosis cerebri, could not be entirely dismissed due to the use of corticosteroids, the symptoms improved with corticosteroid treatment. Post-biopsy imaging studies (Figure 4) showed a significant reduction in perilesional edema following corticosteroid therapy.

During the patient’s hospitalization, dexamethasone was initiated at a dose of 4 mg intravenously every six hours for 10 days. This dosage was then tapered to 4 mg intravenously every eight hours for the following 10 days, and subsequently to 2 mg intravenously every eight hours for an additional 10 days. By day 20 of the admission, the patient demonstrated intact and symmetric cranial nerve function, normal cerebellar testing, and no motor or sensory deficits. The patient was discharged with plans for outpatient follow-up and prescribed oral dexamethasone at a dosage of 0.5 mg once daily orally for five days.

## Discussion

Clinical characteristics

The lack of a pathognomonic feature pointing to a specific diagnosis means we must consider certain information to help us reach a final diagnosis. Glioblastoma and PCNSL are the most frequent causes of butterfly-shaped tumors, followed by demyelinating and infectious diseases [1].

Tumefactive multiple sclerosis is more common in young, female patients. High-grade gliomas have an acute presentation, while tumefactive multiple sclerosis is subacute and chronic [7]. Butterfly gliomas cause symptoms related to disconnection syndromes, including alexia, agraphia, and apraxia, while sensomotoric deficits are more atypical [6]. Generalized tonic-clonic seizures and dysphasia are more related to high-grade gliomas, while ataxia, hemi-sensory disturbance, and diplopia are more common in tumefactive multiple sclerosis [7].

While in immunocompetent patients, the butterfly appearance of a brain tumor is highly suggestive of glioblastoma, PCNSL can also mimic this pattern in immunocompromised patients [9].

The clinical presentation of increased pressure inside the skull due to bleeding from these lesions is usually seen in glioblastomas or metastases [10]. Our case is particularly unique, as there are no reports of butterfly-wing lesions with intratumoral hemorrhage resulting in tumefactive multiple sclerosis.

Imaging studies

Around 60% of butterfly gliomas infiltrate the genu of the corpus callosum, while 30% infiltrate the body and 10% infiltrate the splenium. Frequently, butterfly gliomas result in high-grade glioma diagnosis, usually glioblastoma, which constitutes around 3% of all butterfly gliomas. Infiltration of the corpus callosum may be indicative of an aggressive tumor and a challenging surgery [6]. In our case, we had a lesion associated with intratumoral hemorrhage at the level of the genu of the corpus callosum, which initially raised a strong suspicion of glioblastoma.

By contrast, tumefactive demyelinating lesions on MRI demonstrate a partial or complete ring enhancement [11]. Additionally, perfusion imaging aids in differentiating between tumefactive multiple sclerosis and brain tumors as the former has a relatively decreased cerebral blood volume compared to tumors. However, perfusion images might not be conclusive in PCNSL as it has a decreased cerebral blood volume, similar to demyelinating lesions [12]. Therefore, magnetic resonance spectroscopy (MRS) and cerebrospinal fluid analysis/cytology are useful in differentiating PCNSL from tumefactive multiple sclerosis [12-14]. We decided not to conduct a cerebrospinal fluid study through lumbar puncture due to the high risk of cerebellar tonsillar herniation caused by severe intracranial hypertension observed in imaging studies. Given the urgency of the case, we prioritized biopsy instead of MRS to definitely establish the diagnosis.

Tumefactive demyelinating lesions are typically distinguished from cerebral neoplasms on MRI by their incomplete enhancement, higher number of lesions, minimal to no mass effect and edema, T2 hypointense rim, and generally smaller size. However, some studies suggest that these MRI features are non-specific for tumefactive demyelination. A higher apparent diffusion coefficient, along with MRI and CT hypoattenuation in the contrast-enhancing portions of the lesions, has proven useful in diagnosing tumefactive demyelinating lesions [7]. Our case presented incomplete contrast enhancement, which may be more closely associated with a tumefactive demyelinating lesion.

On MRI, PCNSL is described as T2-weighted iso to hypointense with restricted diffusion. Due to the blood-brain barrier disruption, MRI shows a homogeneous, marked contrast enhancement. Rare findings include hemorrhage, calcifications, necrosis, and cyst formation [15]. The presence of intratumoral hemorrhage is more frequently observed in glioblastoma, metastases, and in some lymphoma cases [10,15]. We did not find any reported cases of tumefactive multiple sclerosis associated with intracerebral hemorrhage in the literature.

Pathology

Although not routinely sampled, when tumefactive demyelinating lesions are biopsied, hematoxylin and eosin stains demonstrate foamy macrophages with reactive gliosis, Luxol fast blue stains show the absence of myelin, glial fibrillary acidic protein stains show reactive gliosis, and neurofilament protein stains show demyelination [7,11].

On the other hand, glioblastomas are classically described as hypercellular lesions with nuclear atypia, cellular pleomorphism, increased mitotic activity, microvascular proliferation (glomeruloid vasculature), and/or necrosis [16]. Likewise, it has been reported that novel vessels may rarely contain multiple Weibel-Palade bodies, which are typically absent in brain endothelial cells. These vessels may exhibit thrombi, causing endothelial damage and proliferation. Two different patterns of necrotic regions are observed: one involves large central confluent areas of necrotic necrosis (i.e., geographic necrosis), while the other consists of tumor cells focally rimming multiple small foci surrounded by central necrosis (i.e., pseudopalisading necrosis). The former is common with primary glioblastoma, while the latter is seen in both primary and secondary types [17]. The molecular profile includes both IDH-mutant and IDH-wild types, which can be identified through immunohistochemistry and molecular testing. Glioblastoma also exhibits positivity for glial fibrillary acid protein, vimentin, and S100, with varying Ki-67 indices [18].

Approximately 95% of PCNSL cases are diffuse large B-cell lymphoma, while the remaining 5% include low-grade B-cell lymphoma, T-cell lymphoma, and Burkitt lymphoma. Diffuse large B-cell lymphoma, not otherwise specified, has two histological subtypes, namely, the germinal center subtype, which is often positive for CD10 and/or BCL6 and negative for MUM1, and the activated B-cell/non-germinal center-like subtype, which is negative for CD10, with or without BCL-6 expression with expression of typically expressing MUM1. Over half of PCNSL cases express both BCL6 and MUM1, indicating that the tumor likely originates from B cells transitioning out of the germinal center but not yet fully at the post-germinal center stage [19-21]. PCNSL typically expresses pan B-cell markers such as CD19, CD20, CD22, and CD79a, but generally lacks plasma cell markers such as CD38 and CD138. While the role of these B-cell differentiation markers is well understood in systemic diffuse large B-cell lymphoma, their importance in PCNSL is still unclear. Similarly, although overexpression of the cell cycle regulator MYC and the anti-apoptosis protein BCL2 has prognostic value in the systemic diffuse large B-cell lymphoma, their significance in PCNLS remains uncertain [19]. PCNSL is characterized by high cellularity and infiltrative behavior. Histological examination frequently reveals a perivascular growth pattern referred to as angiotropism [22]. The presence of a reactive perivascular T-cell infiltrate is associated with a better prognosis, although it is less common in PCNSL than in secondary central nervous system lymphoma. This difference may contribute to the generally poorer prognosis observed in PCNSL [19].

## Conclusions

This case highlights the crucial importance of accurately diagnosing butterfly lesions affecting the corpus callosum to avoid unnecessary and potentially harmful treatments. Advanced imaging techniques such as perfusion MRI and MRS, combined with biopsy, are essential for distinguishing between conditions such as tumefactive multiple sclerosis and malignant tumors such as glioblastoma. Tumefactive multiple sclerosis often responds well to steroid treatment, in contrast to the invasive interventions required for glioblastoma, underscoring the significance of a proper diagnosis. A multidisciplinary approach involving neurologists, radiologists, neurosurgeons, and pathologists is vital for evaluating complex cases, ensuring appropriate treatment, and improving patient outcomes while minimizing risks.
